# 283. Population-Based Co-infection of Carbapenem-Resistant Organisms and SARS-CoV-2 in Massachusetts

**DOI:** 10.1093/ofid/ofab466.485

**Published:** 2021-12-04

**Authors:** Ashley Geoghegan, Jessica Leaf, Monina Klevens, Scott Troppy

**Affiliations:** 1 Office of Integrated Surveillance and Informatics Services, Bureau of Infectious Disease and Laboratory Sciences, Massachusetts Department of Public Health, Jamaica Plain, Massachusetts; 2 Massachusetts Department of Public Health, Jamaica Plain, Massachusetts

## Abstract

**Background:**

Concerns about antibiotic resistance are exacerbated in COVID-19 patients due to frequent antibiotic use, increase in mechanical ventilation and reusable equipment, conservation of PPE, and strain on hospital staff. We described cases with co-infection of carbapenem-resistant organisms (CRO) and SARS-CoV-2 and compared rates in the Massachusetts population.

**Methods:**

All providers and hospitals are required to report CROs and SARS-CoV-2 to the Massachusetts Virtual Epidemiologic Network (MAVEN). We selected cases with both a positive SARS-CoV-2 test and a laboratory confirmed CRO from January through July 2020. We classified by which result occurred first and described demographic and clinical characteristics. We standardized the CRO case definition by excluding CR-*Pseudomonas aeruginosa* and calculated rates per 100,000 to assess the impact of SARS-CoV-2 on the population-based frequency of CROs. Analyses were conducted in SAS 9.4.

**Results:**

28 confirmed cases of SARS-CoV-2 infection were also diagnosed with a CRO. They were an average age of 71.8, 60.7% male, 67.9% white, and 64.3% were in congregate care prior to their diagnoses. Mortality was 5/28 (17.9%). The 23 (82.1%) with a positive SARS-CoV-2 result first were all hospitalized at least once compared to 40% in the CRO first group (p=0.003). 11 (47.8%) of the SARS-CoV-2 first were already admitted when they tested CRO positive; 7 (30.4%) were admitted for the CRO separately from COVID-19 treatment. None of the CRO first group were admitted for CRO infection. Average length of stay for the SARS-CoV-2 first group was higher than the CRO first group (62.3 days vs 11.0 days; p=0.049). Cases positive for CRO first were all infected with CR-*Escherichia coli* whereas those positive for SARS-CoV-2 first were infected with CRAB, CRPA, or a CRE (*Klebsiella oxytoca* or *Klebsiella pneumoniae*) (p< 0.0001). The rate of CRO/COVID coinfection was 0.203 per 100,000 population; the rates for January through July of CRO alone were 2.5 per 100,000 in 2020 and 2.4 per 100,000 in 2019.

**Conclusion:**

Characteristics of individuals co-infected with CRO and SARS-CoV-2 differed by which diagnosis was made first; however, the SARS-CoV-2 pandemic did not impact the CRO population rate during the time frame studied.

**Disclosures:**

**All Authors**: No reported disclosures

